# Supplementary material for: DNAH3 deficiency causes flagellar inner dynein arm loss and male infertility in humans and mice
Source: eLife. 2024 Nov 6;13:RP96755. doi: 10.7554/eLife.96755 (PMC11540302; doi:10.7554/eLife.96755)
Supplement: Figure 1—source data 2. [file elife-96755-fig1-data2.docx]

**Figure 1 — source data 2. Primers for Sanger sequencing.**

| **Target** | **Forward primer (5’—3’)** | **Reverse primer (5’—3’)** | **Product (bp)** |
| --- | --- | --- | --- |
| c.3590C>G  c.3590C>T | TCTGTTATGGAGAAAAGAACCAA | GATGAAAAGTGATAAAAGAGAGTGG | 399 |
| c.4837G>T | TAACGCTGACCCCTGAATCTC | CAAGGTCCTGAACCGCTACAT | 876 |
| c.5587del | GCCCACCATATGGAGAAGAA | GAAATCAAGGGGCAGATGAA | 474 |
| c.10355C>T | TCCCTGGAGATCGAACGTAG | TGTGCCGTTCTCTGTTTGAG | 449 |
| c.2314C>T | CAGTGGGAAACCAAAGGAAA | CACACCACTCCTGTTGACCT | 392 |
| c.4045G>A | CAGAGTCTTCTTCCCCTGGA | TTGGAGAATGGGGGACCT | 368 |
