# Supplementary figures and images for: DNAH3 deficiency causes flagellar inner dynein arm loss and male infertility in humans and mice
Source: eLife. 2024 Nov 6;13:RP96755. doi: 10.7554/eLife.96755 (PMC11540302; doi:10.7554/eLife.96755)

**Figure 1D**

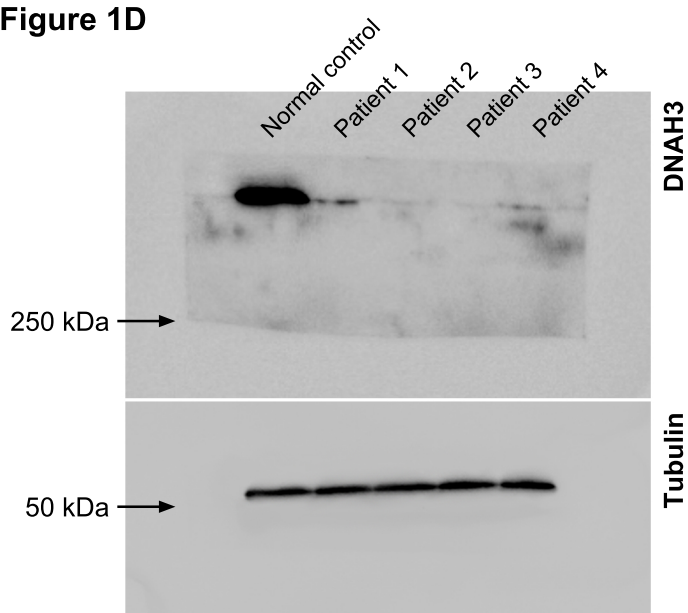

Supplement: Figure 1—source data 3. [file elife-96755-fig1-data3.zip › Figure 1 - source data 3.pdf]

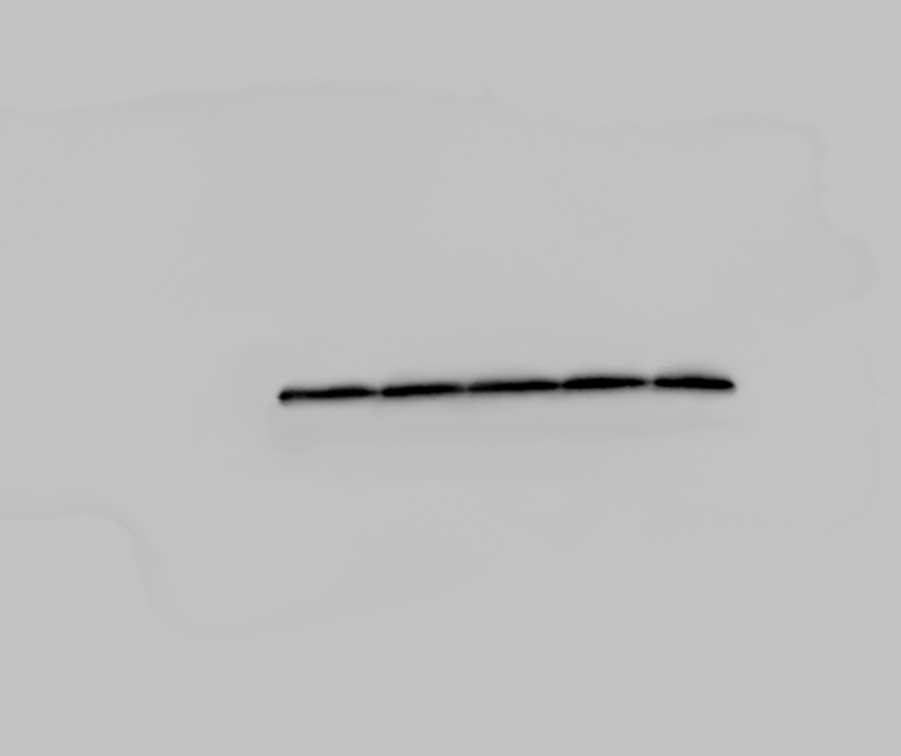

Supplement: Figure 1—source data 4. [file elife-96755-fig1-data4.zip › data 2.tif]

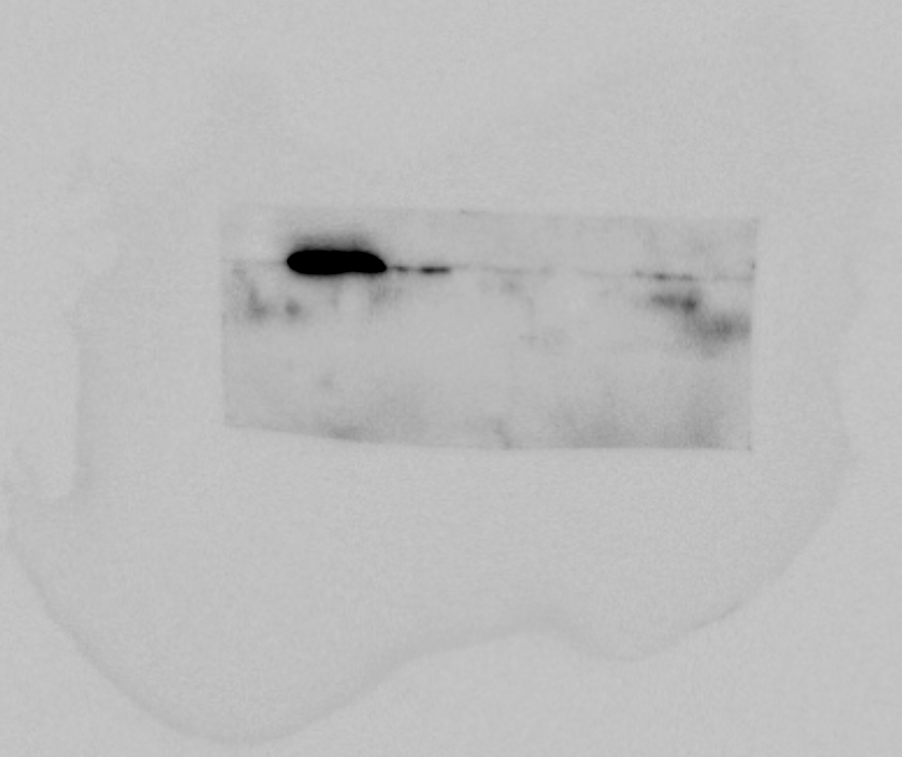

Supplement: Figure 1—source data 4. [file elife-96755-fig1-data4.zip › data 1.tif]

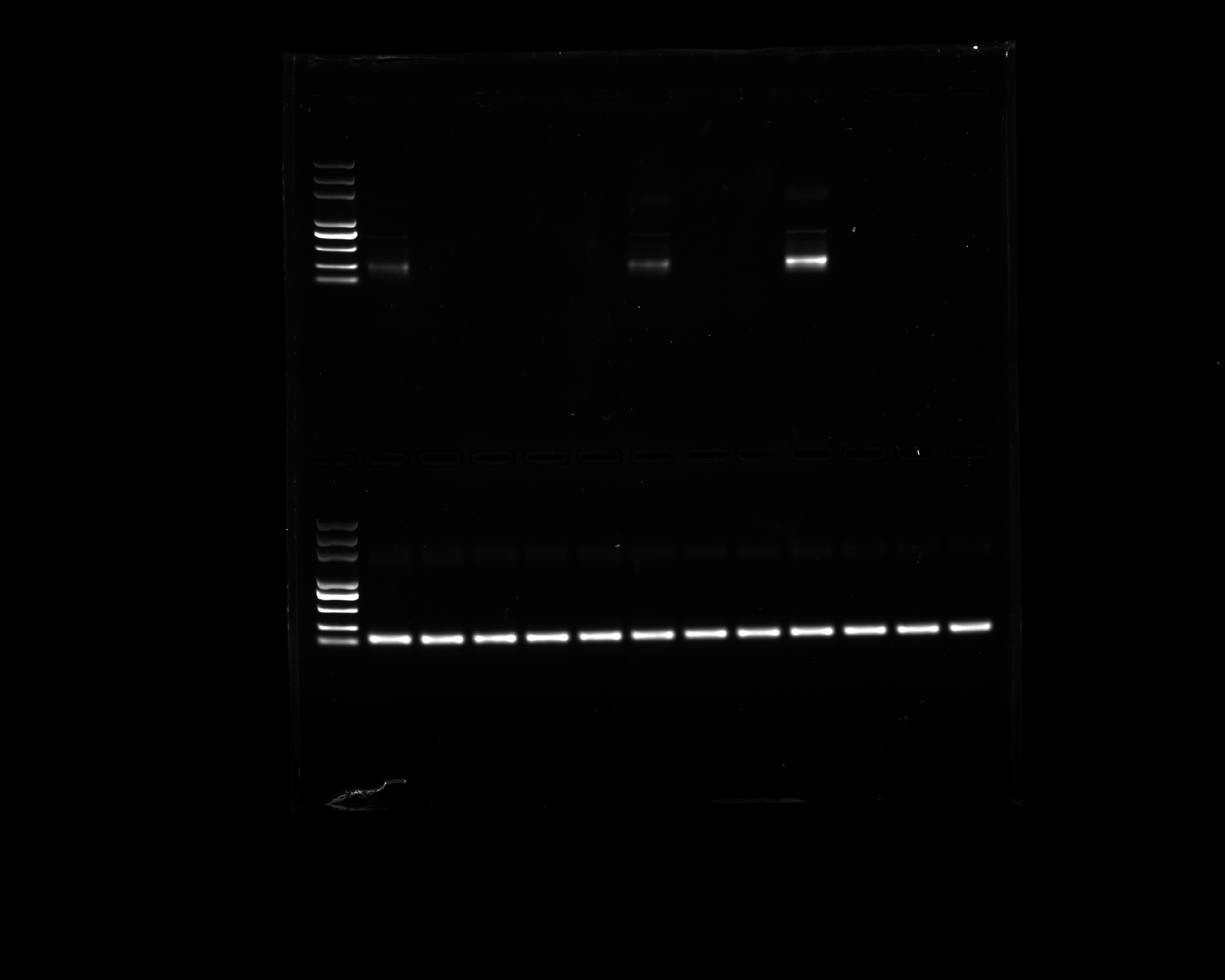

Supplement: Figure 4—figure supplement 1—source data 2. [file elife-96755-fig4-figsupp1-data2.zip › Figure 4 – figure supplement 1A.tif]

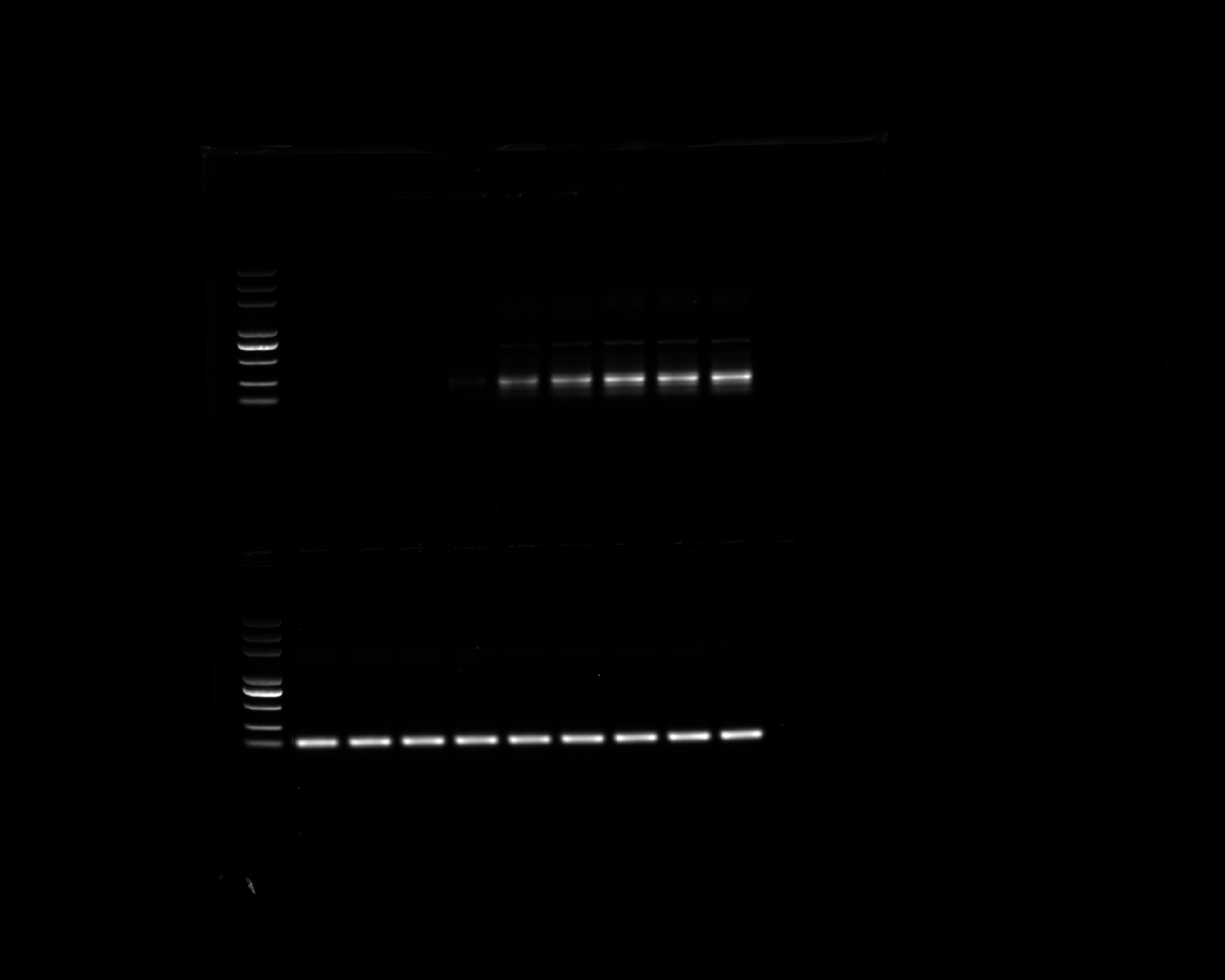

Supplement: Figure 4—figure supplement 1—source data 2. [file elife-96755-fig4-figsupp1-data2.zip › Figure 4 – figure supplement 1B.tif]

**Figure 5D****Figure 6D**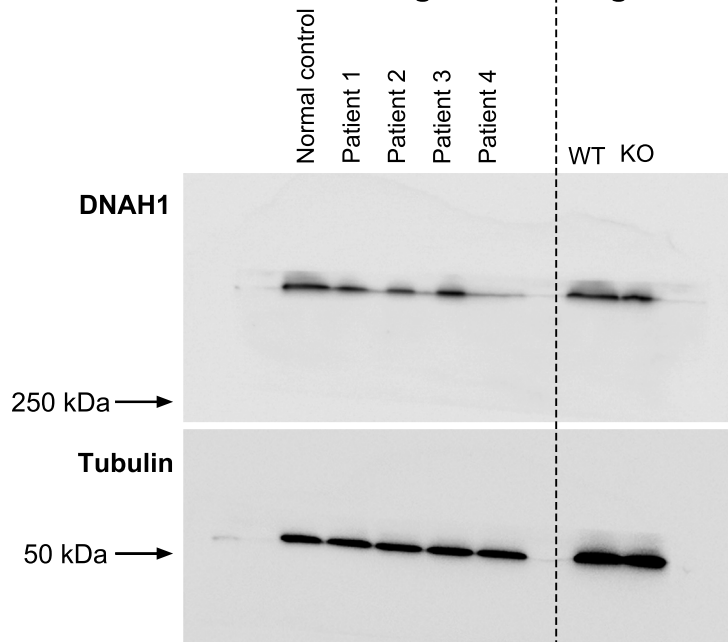**Figure 5E****Figure 6E**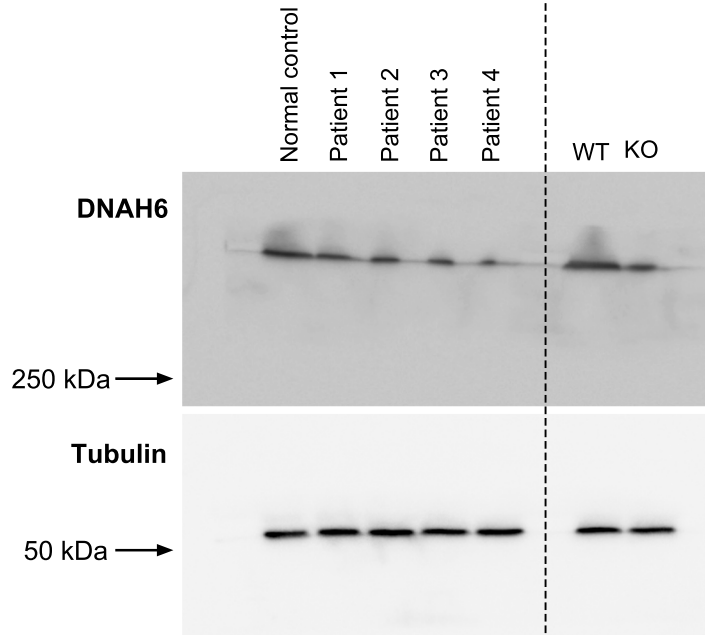**Figure 5F****Figure 6F**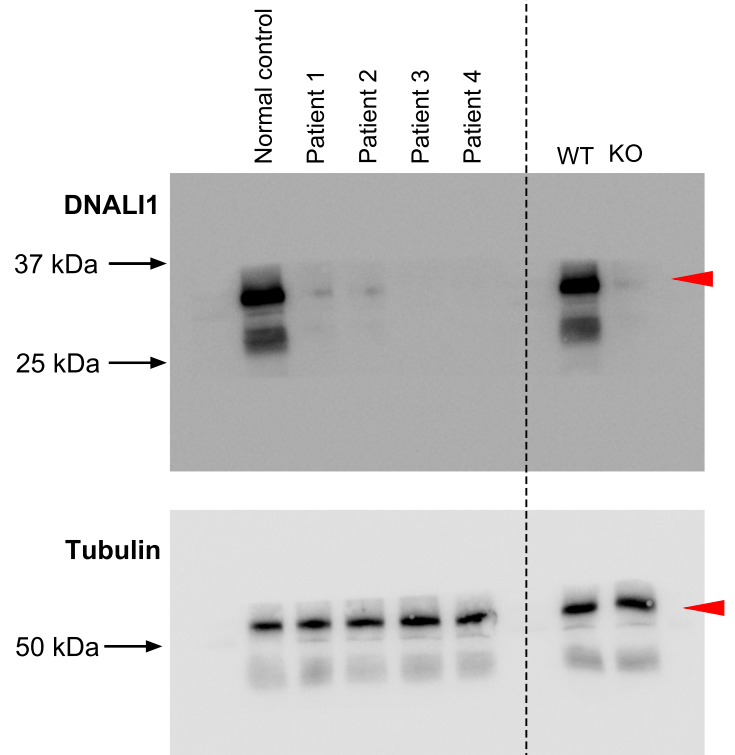

Supplement: Figure 5—source data 1. [file elife-96755-fig5-data1.zip › Figure 5 source data 1.pdf]

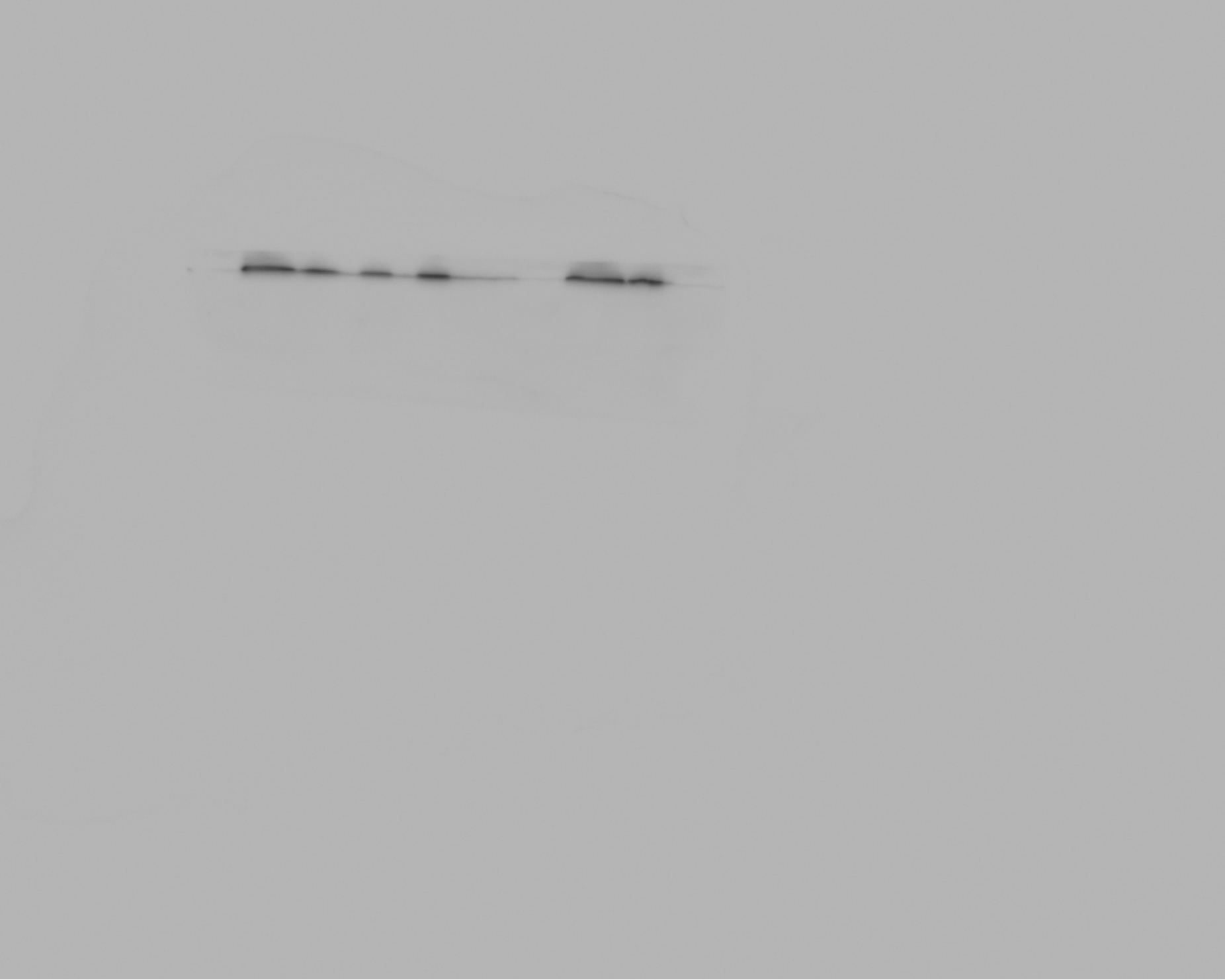

Supplement: Figure 5—source data 2. [file elife-96755-fig5-data2.zip › Figure 5D-Figure 6D/data 1.tif]

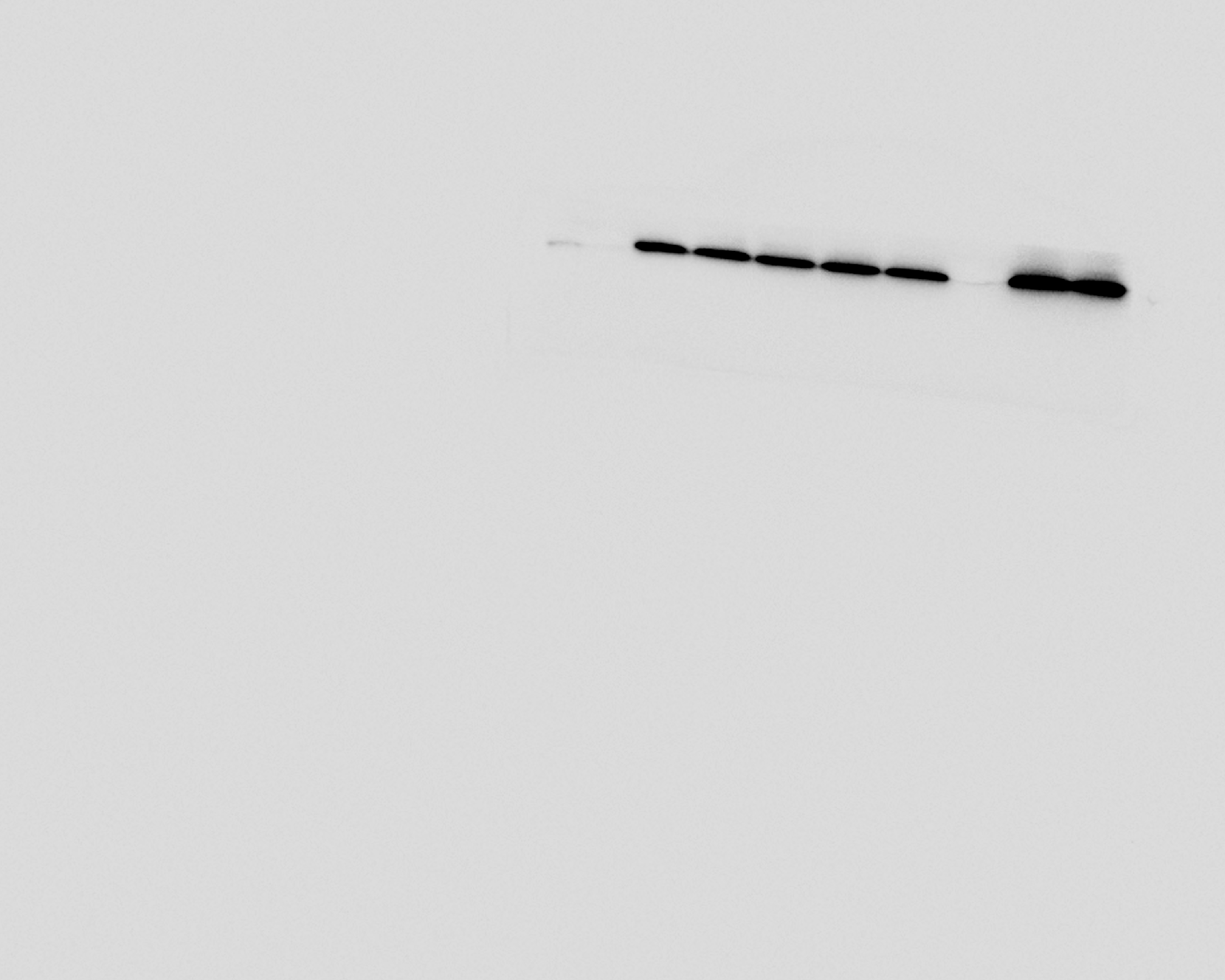

Supplement: Figure 5—source data 2. [file elife-96755-fig5-data2.zip › Figure 5D-Figure 6D/data 2.tif]

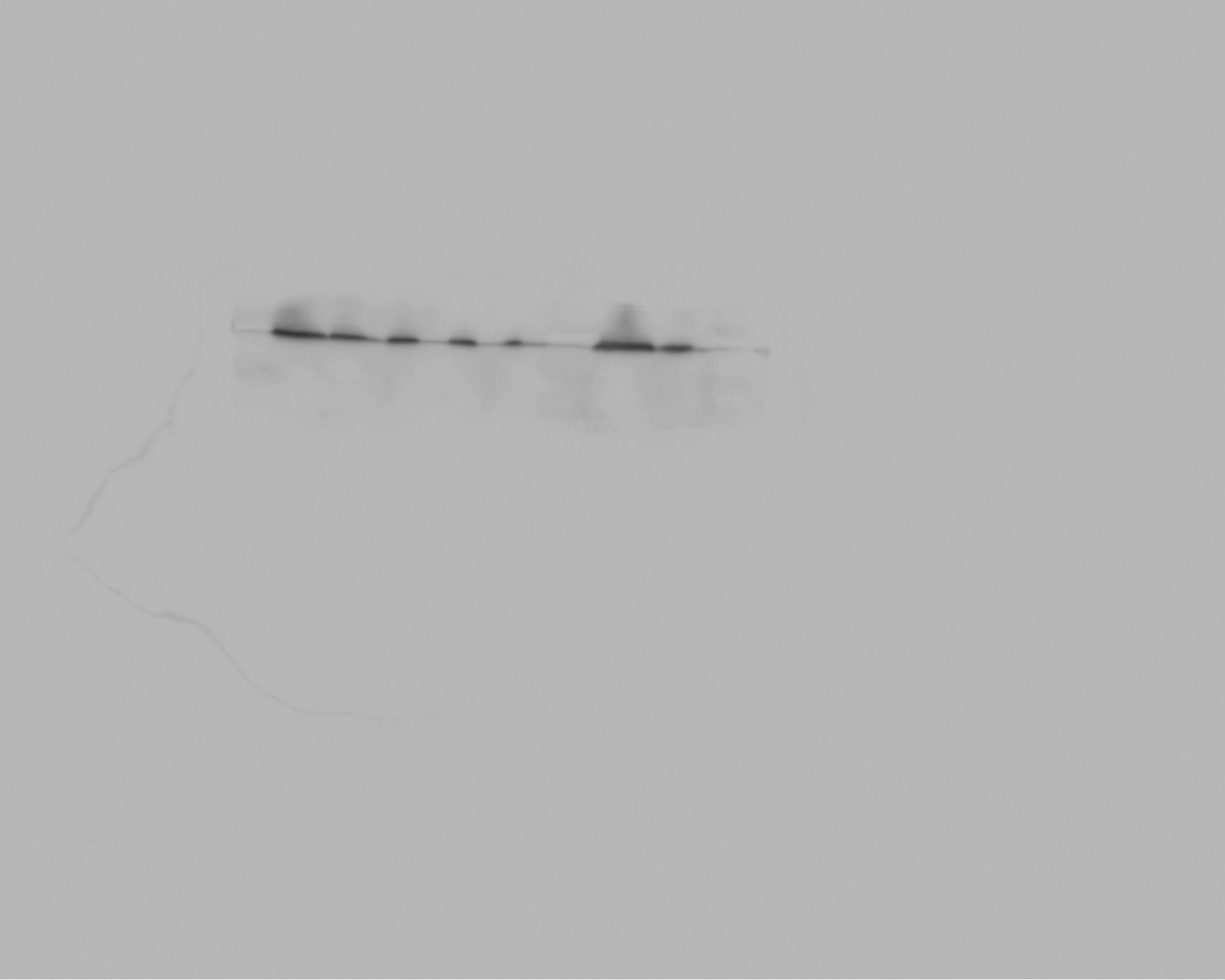

Supplement: Figure 5—source data 2. [file elife-96755-fig5-data2.zip › Figure 5E-Figure 6E/data 1.tif]

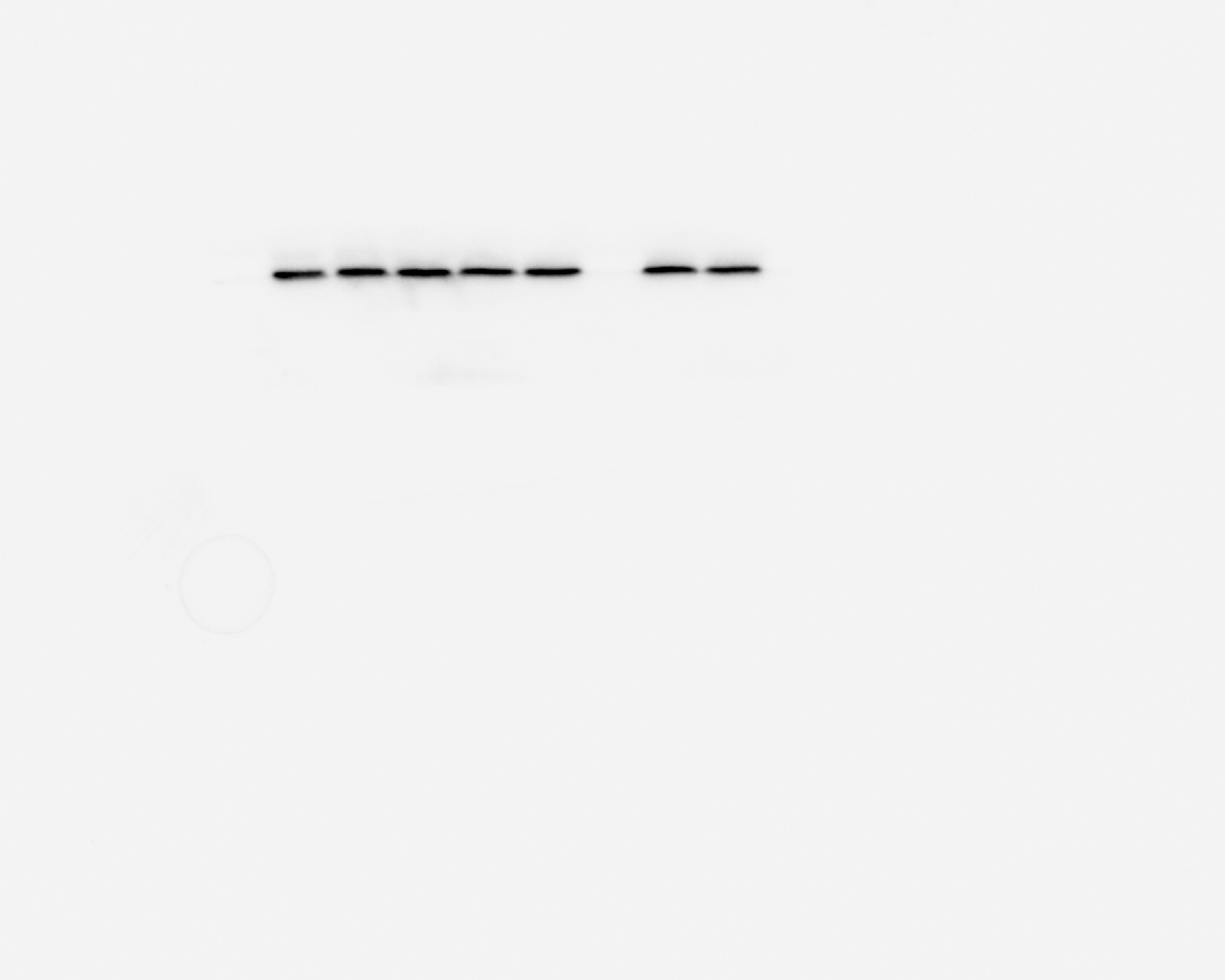

Supplement: Figure 5—source data 2. [file elife-96755-fig5-data2.zip › Figure 5E-Figure 6E/data 2.tif]

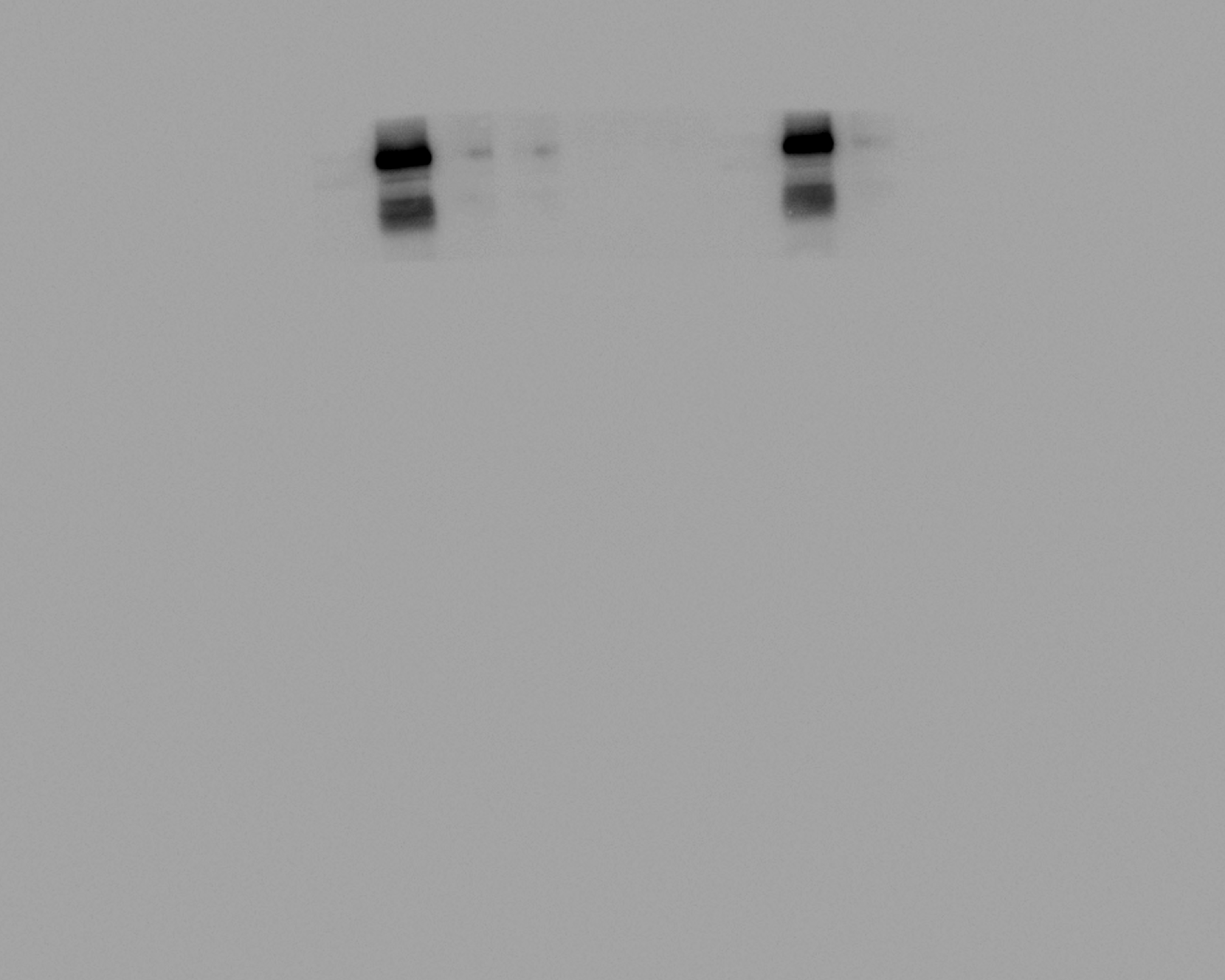

Supplement: Figure 5—source data 2. [file elife-96755-fig5-data2.zip › Figure 5F-Figure 6F/data 1.tif]

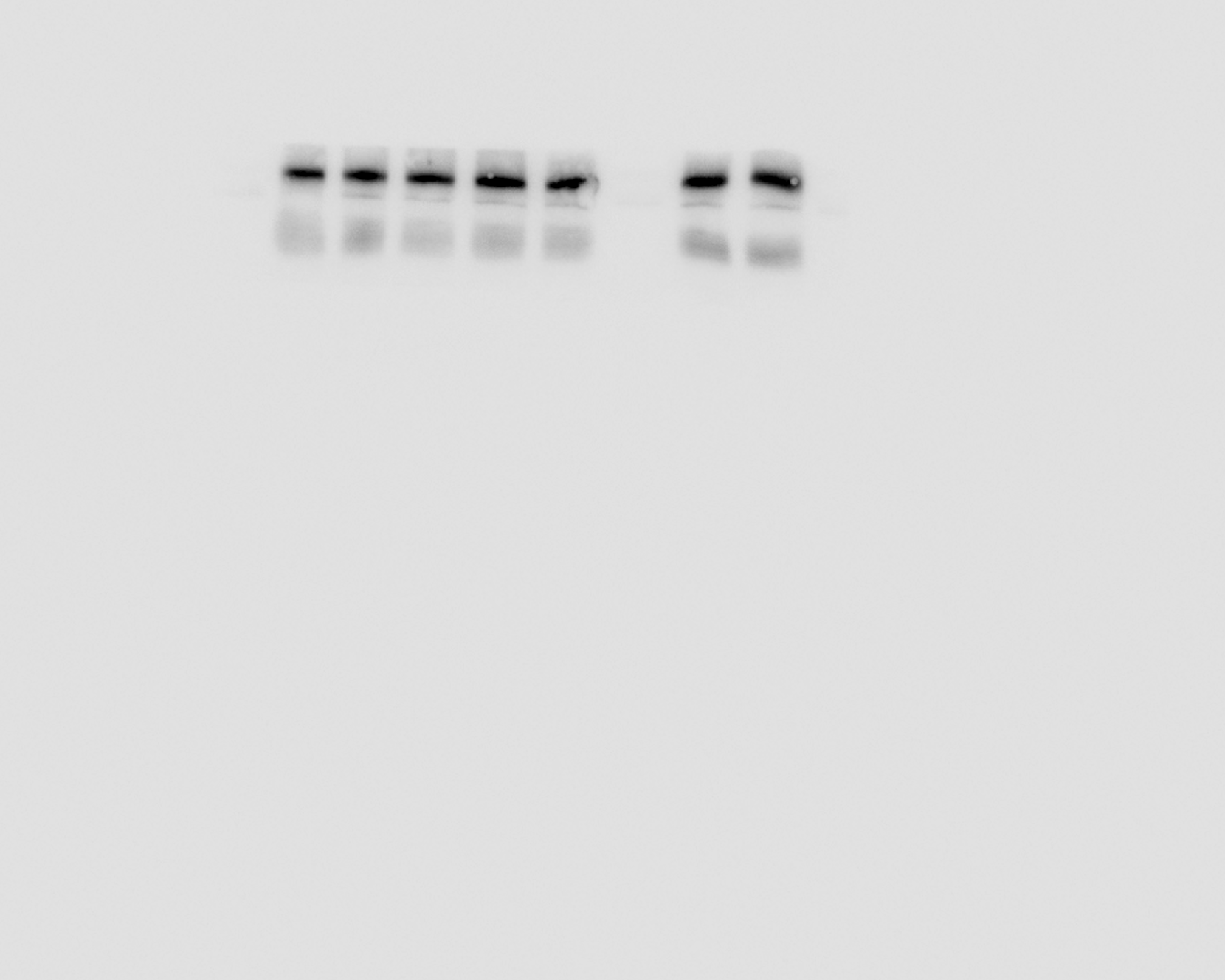

Supplement: Figure 5—source data 2. [file elife-96755-fig5-data2.zip › Figure 5F-Figure 6F/data 2.tif]
