# Supplementary material for: DNAH3 deficiency causes flagellar inner dynein arm loss and male infertility in humans and mice
Source: eLife. 2024 Nov 6;13:RP96755. doi: 10.7554/eLife.96755 (PMC11540302; doi:10.7554/eLife.96755)

Figure 4 – figure supplement 1A

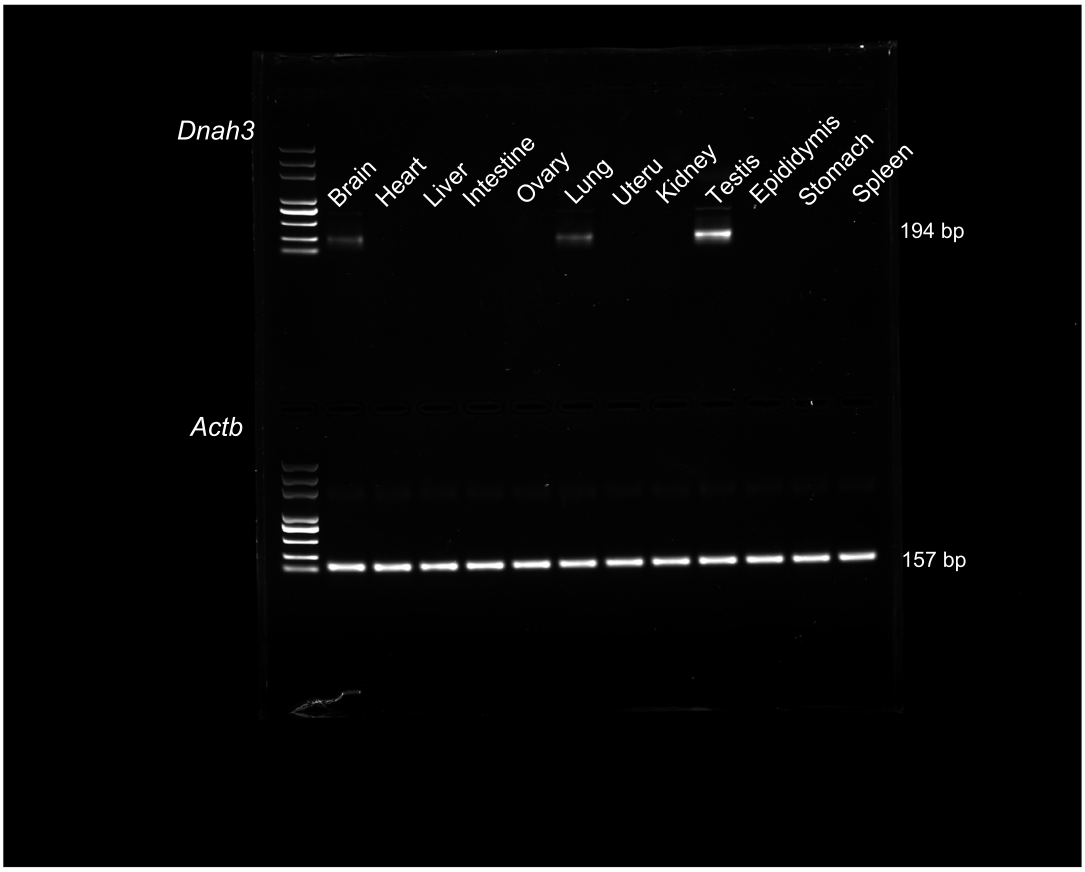

Figure 4 – figure supplement 1B

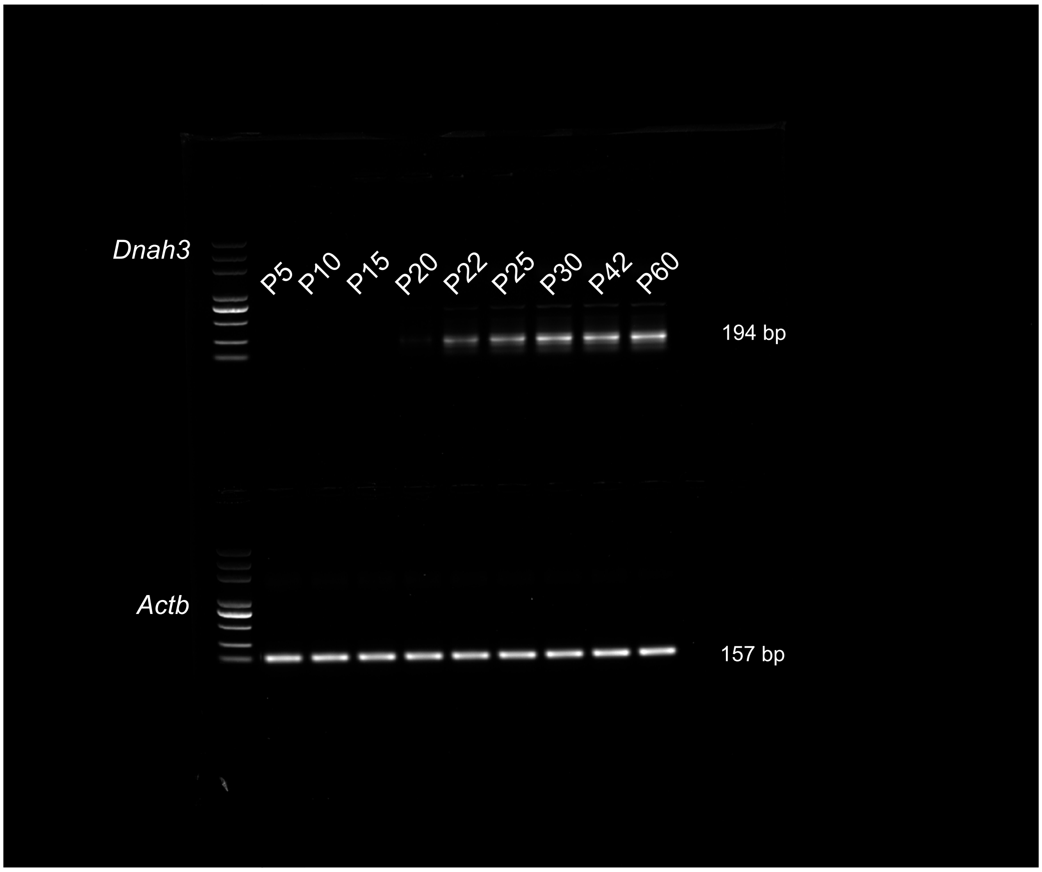

Supplement: Figure 4—figure supplement 1—source data 1. [file elife-96755-fig4-figsupp1-data1.zip › Figure 4 – figure supplement 1 – source data 1.pdf]
