# Supplementary material for: DNAH3 deficiency causes flagellar inner dynein arm loss and male infertility in humans and mice
Source: eLife. 2024 Nov 6;13:RP96755. doi: 10.7554/eLife.96755 (PMC11540302; doi:10.7554/eLife.96755)
Supplement: Figure 4—figure supplement 3—source data 1. [file elife-96755-fig4-figsupp3-data1.docx]

**Figure 4 – figure supplement 3 – source data 1. Primers for Sanger sequencing and qPCR**

| **Target** | **Forward primer (5’—3’)** | **Reverse primer (5’—3’)** | **Product (bp)** |
| --- | --- | --- | --- |
| **Sanger sequencing** | | | |
| *Dnah3* | GAGAAGGGCATCAGTGAATT | TGTGGAGGTCCGTGGTTGAT | 624 |
| **qPCR** | | | |
| *Dnah3* | GGAGGTGATGATGCGAATTT | ATCGAGGGATGCTCTTGATG | 194 |
| *Actb* | CAGCTTCTTTGCAGCTCCTT | CACGATGGAGGGGAATACAG | 157 |
